# Single-cell RNA-seq: advances and future challenges

**DOI:** 10.1093/nar/gku555

**Published:** 2014-07-22

**Authors:** Antoine-Emmanuel Saliba, Alexander J. Westermann, Stanislaw A. Gorski, Jörg Vogel

**Affiliations:** Institute for Molecular Infection Biology, University of Würzburg, Josef-Schneider-Straße 2, D-97080 Würzburg, Germany

## Abstract

Phenotypically identical cells can dramatically vary with respect to behavior during their lifespan and this variation is reflected in their molecular composition such as the transcriptomic landscape. Single-cell transcriptomics using next-generation transcript sequencing (RNA-seq) is now emerging as a powerful tool to profile cell-to-cell variability on a genomic scale. Its application has already greatly impacted our conceptual understanding of diverse biological processes with broad implications for both basic and clinical research. Different single-cell RNA-seq protocols have been introduced and are reviewed here—each one with its own strengths and current limitations. We further provide an overview of the biological questions single-cell RNA-seq has been used to address, the major findings obtained from such studies, and current challenges and expected future developments in this booming field.

## INTRODUCTION

The analysis of single cells by global approaches has the potential to change our understanding of whole organisms since cell lineages can be traced and heterogeneity inside an organ be described with unprecedented resolution (1). Studying cells at the single-cell level offers unique opportunities to dissect the interplay between intrinsic cellular processes and extrinsic stimuli such as the local environment or neighboring cells in cell fate determination. Single-cell studies are also of paramount interest in the clinics, helping to understand how an ‘utlier cell’ may determine the outcome of an infection (2), drug or antibiotic resistance (3,4) and cancer relapse (5). Furthermore, since the vast majority of living cells in the environment cannot be cultivated *in vitro* (sometimes referred to as ‘microbial dark matter’ (6)), single-cell approaches hold the promise of discovering unknown species or regulatory processes (6) of biotechnological or medical relevance.

Global studies of single cells have been enabled by a tremendous increase in the sensitivity of scientific instruments and an ever-growing automation of all steps from sample preparation to data analysis. Nowadays, one can rapidly sequence the genomes of many single cells in parallel using next-generation sequencing techniques (7), or profile expressed proteins using fluorescence and mass cytometry (8). mRNA profiling of single cells has been pioneered by a host of probe-dependent methods including reporter fusions to fluorescent proteins, fluorescence *in situ* hybridization (FISH), quantitative real-time PCR (qRT-PCR), and microarrays (9), some of which can report expression changes of multiple genes in parallel. In this review, we will focus on the analysis of single-cell transcriptomes by RNA-seq, a technique that has already revolutionized the scope and depth of transcriptome analysis of cell populations.

The transcriptome constitutes an essential piece of cell identity since RNA plays diverse roles as a messenger, regulatory molecule, or essential component of housekeeping complexes. Genome-wide transcriptomics, ideally profiling all coding and non-coding cellular transcripts, is therefore well suited to reveal the state of a cell in a specific environment. The probe-independent RNA-seq technique (10,11), in which cellular RNA molecules are converted into cDNA and subsequently sequenced in parallel using next-generation sequencing technology (7), is increasingly becoming the method of choice to achieve this task. Importantly, it can cover the entire transcriptome with single-nucleotide resolution, a feat that is practically impossible to achieve with any of the previous gene expression profiling techniques. Genome-wide RNA-seq analyses have recently uncovered an unexpected complexity in the transcriptomes of organisms from all domains of life with respect to gene structure and output from non-coding regions (12–27). It is now clear that eukaryotic genomes are pervasively transcribed; for example, while protein-coding genes constitute less than 2% of the human genome, more than 80% of its regions may be transcribed (13). In addition, many genomic loci give rise to multiple transcripts, and this has dramatically changed our perception of genome organization, the definition of a gene and the diversity of functions exerted by RNAs (28–31). Likewise, RNA-seq has facilitated the annotation of prokaryotic genomes by defining 5′ and 3′ untranslated regions of mRNAs and discovered many previously unrecognized RNA molecules including an unexpected degree of genome-wide antisense transcription (21). Moreover, variants of the RNA-seq technique globally determine many other RNA-related aspects in the cell, for example, secondary structures of transcripts (32), editing sites (33), transcript stability (34), translation rates (35) and the protein–RNA interactome (36).

To date, most transcriptome studies are conducted on a ‘population level’ usually averaging the transcriptomes of millions of cells. However, in some cases such as stem cells, circulating tumor cells (CTCs) and other rare populations, sufficient material cannot be obtained for analysis on such a scale. In addition, bulk approaches fail to detect the subtle but potentially biologically meaningful differences between seemingly identical cells. That is, although individual mammalian cells are estimated to contain 10^5^–10^6^ mRNA molecules (37), the relative proportions of different transcript classes in a population are highly variable (38): a quantitative analysis in yeast (39) has shown that the majority of mRNAs are present in a few (<5 transcripts) copies per cell, and most long non-coding RNAs (lncRNAs) even in <0.5 copies per cell. As for bacteria, the average copy number of an mRNA in *Escherichia coli* is 0.4 per cell (40). Furthermore, a specific transcript will be expressed at different levels within a cell population either due to deterministic reasons because it is part of an activated cellular process or due to random different levels of expression between cells, a phenomenon also called transcriptional noise that cannot be considered insignificant since it has broad implications in cell fate decisions (41).

Pioneering single-cell studies of differential gene expression within a cell population in the cellular response to a specific signal or environment mainly relied on fluorescence microscopy techniques whereby only a few genes could be studied simultaneously (42). RNA-seq of single cells has provided the first characterization of the extent of transcriptional differences of both coding and non-coding RNAs on a genome-wide scale (43). In addition to differential gene expression levels, additional layers of transcriptional differences emerge between individual cells. We have learnt that splicing patterns (43) and allelic random expression (44) are widely variable between cells. Single-cell transcriptomics will also help to reconstitute temporal transcription networks during developmental processes (45) or when cells are exposed to external stimuli (43), all of which can be masked on a population level.

As will be reviewed in the next two sections, single-cell RNA-seq requires the successful combination of two independent techniques: the isolation of individual cells of interest from culture, tissue or dissociated cell suspensions, and—after converting the minute amount of cellular RNA into cDNA—the massively parallel sequencing of cDNA libraries. The third and fourth part of this review will discuss current applications and future challenges, respectively, of single-cell RNA-seq.

## ISOLATION OF SINGLE CELLS

The initial step in obtaining the transcriptome of a single cell is the isolation of individual cells from a potentially heterogeneous population. This section provides an overview of the available isolation methods that are compatible with downstream RNA-seq analysis.

### Single-cell isolation from dissociated cell suspensions

#### Flow-activated cell sorting

**Figure 1.**
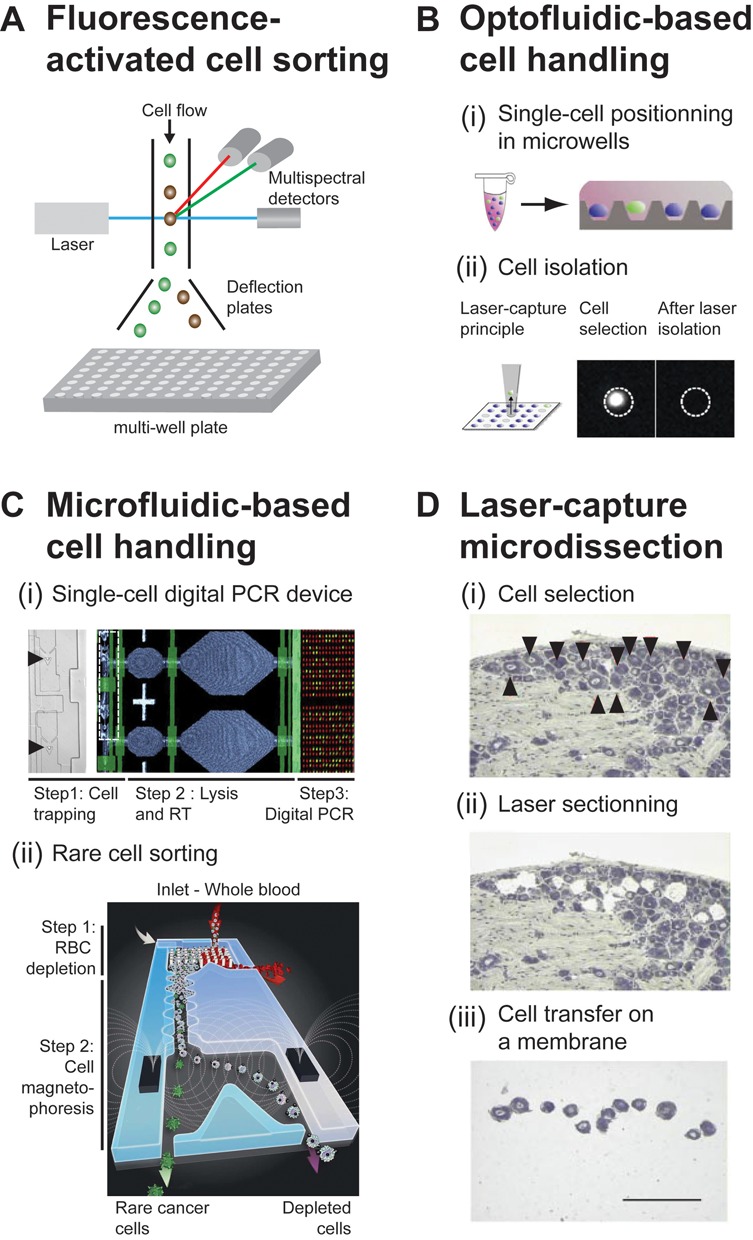
Methods to isolate single cells. (**A**) Principle of fluorescence-activated cell sorting. A stream of droplets, each containing a single cell, passes through an excitation laser beam and the fluorescence signal emerging from each cell is analyzed by a multispectral detector. If the emitted fluorescence signal gates in the preset window, the respective cell will be sorted into a multi-well plate. (**B**) Optofluidic-based cell handling. (i) A cell suspension is arrayed in a plate, each well containing a single cell. (ii) A laser, also called an optical tweezer, is used to manipulate each individual cell. Adapted from (62). Reprinted by permission from Macmillan Publishers Ltd on behalf of Scientific Reports, copyright 2013. (**C**) Two examples of emerging microfluidics-based cell sorting technologies. (i) A microfluidic system integrates all steps from single-cell trapping to gene expression analysis: (step 1) cells are retained individually in microfabricated chambers (each cell is indicated with a black arrowhead); (step 2) cells are lysed and RNA is reverse transcribed; (step 3) detection and analysis is achieved by digital PCR. Adapted with permission from (85,86). Copyright 2013 American Chemical Society. (ii) Representation of a microfluidic system that integrates all steps necessary for sorting extremely rare cancer cells from whole blood: (step 1) red blood cells (RBCs) are separated from white blood cells based on their size and (step 2) rare cells are further isolated from the stream of white blood cells by magnetophoresis. Adapted from (75). Reprinted by permission from Macmillan Publishers Ltd on behalf of Nature Protocols, copyright 2014. (**D**) Laser capture microdissection. (i) Cells of interest are identified in a stained section of rat cervical dorsal root ganglia (indicated by black arrowheads), (ii) cut with a UV laser and (iii) transferred onto a membrane. Scale bar 200 μm. Adapted from (82). Reprinted by permission from Macmillan Publishers Ltd on behalf of Nature Medicine, copyright 1999.

This is the most commonly used method to isolate single cells (Figure 1A); it combines multiparametric flow cytometry and sorting based on a preset fluorescence gating strategy. Fluorescently labeled antibodies are used to isolate cells of interest according to the targeted cell-surface markers. Currently up to 17 individual markers can be used simultaneously (46,47), which enables complex immuno-phenotyping that can identify novel subsets of cells even within previously well-characterized cell populations, for example, a T cell sub-population with stem-cell-like memory and high proliferative capacity (48). The cytometers can be interfaced with 96/384-well plates, allowing hundreds of cells to be efficiently sorted within a couple of minutes to a purity of nearly 100%, one cell per well (49). Furthermore, the ‘index-sorting’ option enables the retrieval and association of the original fluorescent signal with each sorted cell. The popularity of flow-activated cell sorting (FACS) stems mainly from the wide availability of robust commercial platforms within laboratory facilities, their ‘user-friendly’ interfaces, efficient data visualization tools as well as their low running costs.

Another variant of flow cytometry uses antibodies against certain intracellular proteins to select cells according to their signaling state (8,50). As this requires permeabilization and fixation of cells (8,50), it may hamper subsequent transcriptomic analysis. However, a mild fixation does not seem to compromise RNA quality and downstream cDNA synthesis for global transcriptomic investigations (51). Recent progress using the cell cytometry (52) has enabled the isolation of cells based on a defined transcriptional state by quantifying the expression level of a selected transcript labeled by FISH, although the compatibility with downstream single-cell RNA-seq protocols remains to be demonstrated.

Potential limitations of FACS include the need for antibodies that target specific proteins; fortunately, large-scale projects such as the Human Protein Atlas are continuously producing antibodies that will enable the isolation of ever more subtypes of cells (53). Another relevant limitation of FACS is the requirement of a large starting volume, which hampers the isolation of cells from extremely low volume samples (containing a few microliters) such as fine-needle aspirates (54). Similarly, FACS is neither well suited for the isolation of extremely rare cells (for example, one target cell within 1 million non-targets) because of false positive signals, nor for environmental samples containing extremely heterogeneous cell sizes. Currently, FACS also fails to image the cell to be sorted, thereby preventing the combination of morphological and transcriptomic analyses. Developments in flow imaging-based cytometry (55,56) may address this problem in the future.

#### Micromanipulation

Here, a glass micropipette is used to aspirate single cells from a cell population under a microscope. Micromanipulation has been successfully used to pick individual cells such as neurons from rat primary neuronal cultures (57,58), single cells from diverse developmental stages of the embryo (59) as well as individual bacteria (60). Potential limitations of this technique are the significant effort of manual handling and a low throughput (a few cells per hour). Similar to FACS, it can also not be used to manipulate cells present in low microliter volumes.

#### Optical tweezers

Unlike micropipettes, optical tweezers use a highly focused laser beam to physically hold and move microscopic dielectric objects. Combined with imaging-based cell selection, they can trap and manipulate individual cells in suspension (61) or from a cell array inside a microfluidic device (62–65) (Figure 1B). Although currently confined to a few specialized labs due to the demanding optical set-up (65), optical tweezers may soon be available as part of automated robots (62).

#### Microfluidics and other emerging isolation technologies

The rapid expansion of microfabrication techniques and their transfer to biological laboratories has resulted in the first fully integrated microsystems (66) that are able to perform all the steps from cell culture, single-cell isolation to the biochemical steps of cDNA synthesis and detection, an example of which is shown in Figure 1C. Nanoliter microfluidic chambers have been used to isolate non-culturable cells from small-volume microbial community samples for individual genome amplification (64,67). Of note, nanoliter-scale volumes substantially reduce external contamination (68). Beyond microbes, the Fluidigm C_1_^TM^ machine now enables the manipulation of up to 96 mammalian cells in parallel.

Recent advances in engineering have allowed researchers to go beyond using molecular markers by specifying cells based on physiological and biophysical features such as cell size (69), deformation (70,71), and electric (72) or magnetic properties (73). Identifying cells via these novel biomarkers can help isolate cancer (74) and stem (71) cell sub-populations and, combined with single-cell transcriptomics, has the potential to improve our understanding of the underlying inter-cellular differences.

Highly diluted, rare species such as CTCs, of which only a few are present among a million blood cells, constitute a great challenge for cell isolation. CTCs have been isolated from patients using epithelial cell surface markers and microfluidics-based technologies; these technologies have also enabled manipulation of single CTCs (75,76) that in principle could be analyzed via single-cell sequencing (Figure 1C). Together, these systems have enabled the molecular characterization of sub-populations of isolated cells (77,78) and have also been transferred to the clinics. Unfortunately, the use of microfluidics-based techniques has been hindered by the necessity to engineer the devices, which requires specialized equipment and knowledge, their relatively low throughput compared to flow cytometry-based sorting (96 cells treated in parallel against thousands for flow cytometry) and their current high cost, even though the latter can be expected to drop in the future.

### Single-cell isolation from tissue samples

Monolayer cultures of immortalized cell lines have provided valuable *in vitro* models for single-cell gene expression studies, but it has become increasingly clear that long-term passaging of cells can cause dramatic genomic rearrangements and mutations compared to the reference genome (79). This also extends to changes in gene expression, as shown by a comparison of 2D monolayers with 3D spheroid cultures of melanoma cells (80). Moreover, mechanical forces that are present within tissues have a dramatic effect on the expression of many genes (81). As the interest in analyzing primary cells directly obtained from tissue grows, isolation procedures that preserve RNA integrity despite the necessary embedding, fixation, histology staining and cell dissociation procedures are needed. This may be achieved by a combination of tissue cryo-preservation, histological staining and single-cell dissociation with infrared-based microdissection (82,83). Laser-capture microdissection (84), as illustrated in Figure 1D, lends itself to retrieving single cells from a whole tissue. It works without prior dissociation of the cells and thus preserves their 3D structure. An exciting development are *ex vivo* systems that mimic conditions of a cell's local microenvironment, as achieved for complex organs such as the brain, the lung or blood vessels (66). Such reconstituted systems are compatible with microscopy techniques, meaning that they can open new opportunities to combine imaging techniques with single-cell transcriptomics.

## SINGLE-CELL RNA-SEQ

Until recently the method of choice to study gene expression of single cells was multiplexed qRT-PCR; however, its throughput has remained limited to several hundreds of genes even when using highly parallel microfluidic systems (85–87). More fundamentally, qRT-PCR is biased toward the specific set of genes chosen by the experimentalist, and therefore must be hypothesis-driven. Microarrays enable single-cell analyses on transcriptome-wide scale (88–90), but compared to RNA-seq, they suffer from limited sensitivity and dynamic range. In addition, hybridization-based methods typically require large starting amounts of RNA; i.e. microgram quantities (89) versus nanogram quantities required for library preparation for RNA-seq (91,92). Finally, the necessary number of specific probes for a *full* transcriptome coverage, in which non-coding regions are also covered and splice-junctions or processing sites can be defined, makes microarrays a very expensive technology. Thus, as seen previously with populations of cells (11), RNA-seq is also replacing hybridization-based methods on the single-cell level (93).

**Figure 2.**
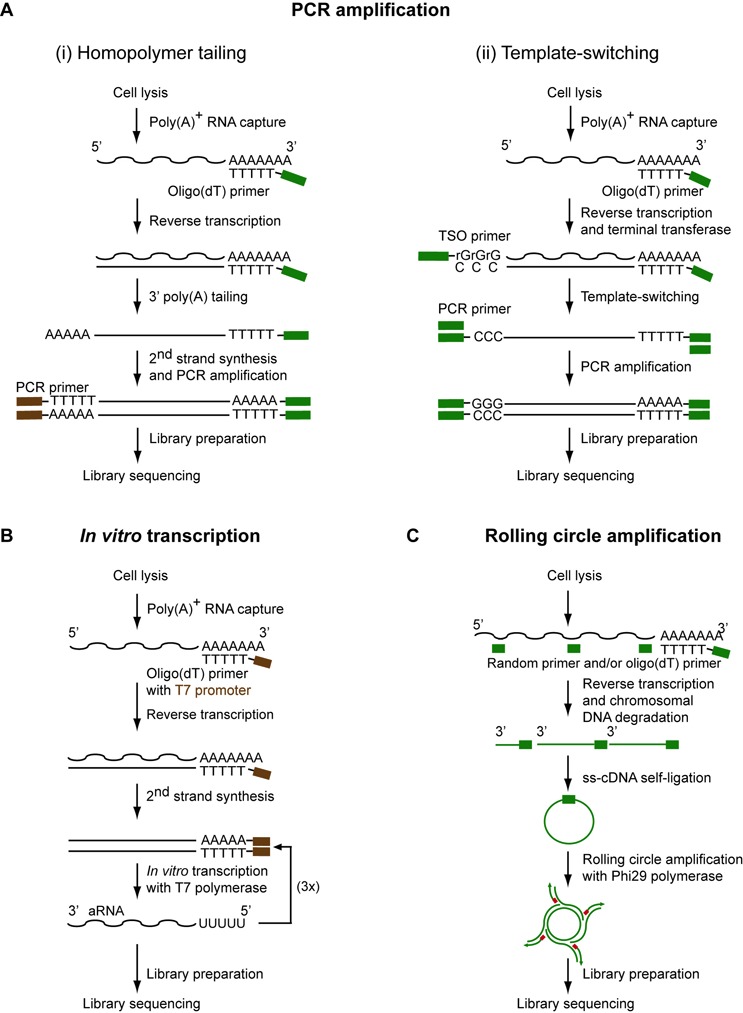
Existing methods to prepare sequencing libraries from a single cell. The methods differ with respect to the strategy used to amplify cDNA that is obtained by oligo(dT)- or/and random-primed reverse transcription. (**A**) Universal primers are inserted at the 3′ end along with the reverse transcription initiation oligonucleotide and at the 5′ end of the initial RNA molecules after reverse transcription via either (i) poly(A) tailing at the 3′ end of the first cDNA strand or (ii) template-switching mechanism involving a template-switching oligonucleotide (TSO). (**B**) During cDNA synthesis, a T7 RNA polymerase primer can be integrated that enables the further amplification of RNA via *in vitro* transcription. This results in an antisense RNA (aRNA) that can be subjected to library preparation and sequencing. (**C**) Alternatively, after cDNA synthesis and chromosomal DNA degradation, cDNA can be circularized and amplified by a technique referred to as rolling circle amplification using the Phi29 polymerase.

Since it is not yet possible to directly sequence RNA molecules, a common strategy used to capture the single-cell transcriptome relies on three major steps (Figure 2): RNA reverse transcription into first-strand cDNA, second-strand synthesis and cDNA amplification, and cDNA sequencing using next-generation sequencing technologies. Since single-cell RNA-seq provides an indirect representation of the transcriptome, a careful assessment of all aspects of the process is required, for example, biological variability versus technical variability, the latter of which is due to loss of specific transcripts during RNA isolation and library preparation; inclusion of different transcript classes; transcript coverage; maintenance of strand specificity; maintaining the initial transcript abundance. Moreover, for statistical significance, one must sequence many individual cells from a given sample (ideally, hundreds to thousands), which makes automation of the entire process desirable. In this section, we will provide an overview of the strategies used to capture RNA and amplify cDNA and discuss the limitations and the strengths of these methods.

### The many routes from single cells to cDNA libraries

#### Cell lysis and reverse transcription

Generally, eukaryotic cells are lysed in a hypotonic buffer containing a detergent. Cells contain many diverse RNA molecules and the gold standard would be to amplify all of them with the exception of tRNA and rRNA that would otherwise populate >90% of the sequencing reads (94) if not removed. Therefore, most methods selectively reverse transcribe polyadenylated RNA using a poly(dT) primer (Figure 2). This poly(A)+ selection strategy has the advantage of capturing the most informative transcripts such as mRNAs and most lncRNAs, while excluding the undesirable rRNAs and tRNAs in a single step. Nevertheless, certain non-polyadenylated yet informative RNAs including microRNAs and non-polyadenylated lncRNAs will be lost (95). Also note that such a strategy is not compatible with RNA isolated from prokaryotic cells since only a minority of the cellular RNAs are polyadenylated, and these represent transcripts that are targeted for degradation (96). To remedy this, 5′-monophosphate-dependent exonuclease has been used to deplete tRNA and rRNA from the lysate of a single prokaryotic cell (97), but this treatment will inevitably also deplete physiologically relevant processed mRNA or small RNA species. Other depletion methods have been proposed for bacteria (94) but a systematic assessment has yet to be conducted.

Subsequently, first-strand cDNA synthesis (Figure 2) is performed using an engineered version of the reverse transcriptase (RT) of Moloney Murine Leukemia Virus (M-MuLV) that has low RNase H activity (98), increased thermostability (99) and produces a higher cDNA yield than other RT enzymes (100). This enzyme enables the generation of RNA:DNA hybrid molecules with an average length of 1.5–2 kb (101).

To generate the second cDNA strand several different protocols have been described based on one of three currently available amplification methods (Figure 2): PCR-based amplification, *in vitro* transcription or rolling circle amplification.

#### PCR-based amplification

This method involves the addition of a universal primer to the cDNA at the 5′ end of the original transcript either after homopolymer tailing of the cDNA or a template-switching reaction. Homopolymer tailing (Figure 2A and Table 1) uses a terminal deoxynucleotidyl transferase to add a ∽30 nt poly(A) stretch (102) to the 3′ end of the first-strand cDNA. The initial protocol stems from the late 1980s (103) and was later optimized for microarray analysis (90,104) and subsequently adapted to RNA-seq (93,105–107). However, this approach has two major problems. First, the premature termination intrinsic to reverse transcription significantly reduces transcript coverage at the 5′ end of transcripts (108). More importantly, the introduction of a poly(A) tail at the 3′ end of the first-strand cDNA in addition to the natural poly(A) sequence at the 3′ end of the input RNA causes a loss of strand information in the resulting double-stranded cDNA molecules.

**Table 1.** Principal characteristics of currently used single-cell RNA-seq methods

	Poly(A) tailing	Template switching	*In vitro* transcription	Rolling circle amplification	5′ selection	3′ selection
Associated acronyms	n/a	SMART-seq	n/a	n/a	STRT	CEL-seq, MARS-seq
Full-length transcripts?	Yes	Yes	Yes	Yes	No	No
Strand specificity?	No	Possible^a^	Possible^a^	No	Yes	Yes
Early pooling^b^?	No^a^	No^a^	No^a^	No	Possible	Possible
Positional bias?	Weakly 3′	Weakly 3′	Weakly 3′	No	5′ only	3′ only
Applied for which cells?	Eukaryotic	Eukaryotic	Eukaryotic	Eukaryotic and prokaryotic	Eukaryotic	Eukaryotic
Key references	(93,105)	(91,108–109)	(57,110)	(97,111)	(37,112)	(113–115)		

n/a: not available. ^a^ would be possible if coupled to long-read sequencing but not with short-read sequencing. ^b^ refers to the possibility to introduce a cellular barcode identifier during first-strand synthesis.

To guarantee homogenous transcript coverage, a template-switching mechanism was developed (108–109,116) (Figure 2A and Table 1). This SMART-seq method (*S*witching *m*echanism *a*t 5′ end of the *R*NA *t*ranscript) utilizes an intrinsic property of RT M-MuLV to add three to four cytosines specifically to the 3′ end of the first cDNA strand, which is subsequently used to anchor a universal PCR primer (117). This ensures that only full-length transcripts are amplified and maintain strand specificity due to the added cytosines (see below). One drawback of template switching, however, seems to be a lower sensitivity compared to homopolymer tailing (107), which may be explained by an imperfect efficiency of RT M-MuLV to add 3′ cytosines.

#### In vitro transcription

This alternative method to amplify first-strand cDNA (Figure 2B) was originally introduced in the early 1990s (110). Also known as the Eberwine method, it was recently adapted to single-cell RNA-seq (113–114,118). Instead of exponential PCR amplification, *in vitro* transcription (IVT) amplifies RNA linearly using T7 RNA polymerase. IVT is biased toward the 3′ end of input transcripts (57), and each of RNA amplification round leads to a further shortening of the transcript occurring during the second strand synthesis (57,119). Several improvements and variants of the method have been developed, and reviewed elsewhere (89). Nonetheless, the IVT protocol remains labor intensive (57). To compensate for this drawback, methods to pool cells and libraries, known by the acronyms CEL-seq (*C*ell *e*xpression by *l*inear amplification and *seq*uencing) (113) and MARS-seq (*Ma*ssively parallel *R*NA single-cell *seq*uencing) (114), have been recently developed (Table 1).

#### Rolling circle amplification

This third strategy has been successfully applied to generate cDNA libraries from single eukaryotic (111) and prokaryotic cells (97). Here the RNA is reverse transcribed, circularized and amplified using Phi29 DNA polymerase (Figure 2C) which preserves full-length transcript coverage. Interestingly, one of these studies (97) has used random primers to generate cDNA, making the approach suitable for prokaryotes.

### Maintaining full-length and strand specificity information

**Figure 3.**
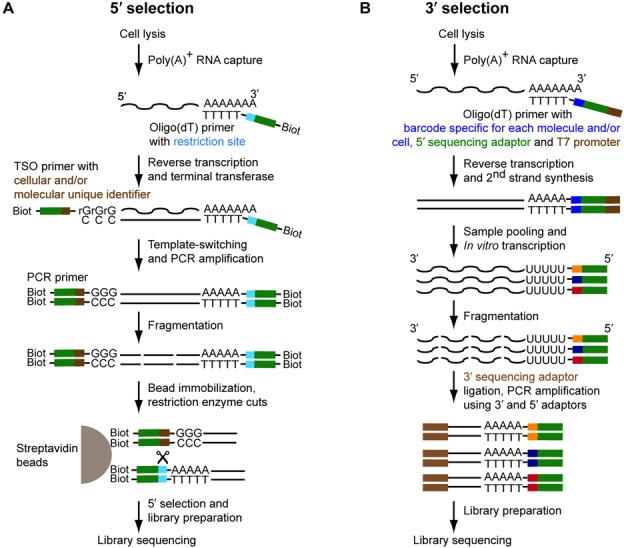
Strand specificity and barcoding strategies. (**A**) Using a template-switching mechanism, a restriction site together with the universal priming sequence can be integrated at the 5′ end of the initial RNA transcript. Likewise biotin can be introduced at both the 3′ and 5′ ends via the use of biotinylated primers. Upon binding to streptavidin beads, enzymatic cleavage will lead to the selection of only the 5′-most fragments for library construction. In addition, during template switching cellular and/or molecular barcodes can be integrated. (**B**) Alternatively using *in vitro* transcription, 3′ ends can be selected after ligation of 3′ and 5′ adaptors to fragmented RNA in order to selectively prime PCR amplification of only the 3′-most fragments. The oligo(dT) primer is used to introduce cellular and/or molecular barcodes.

Given the prevalence of pervasive and antisense transcription (30) it is critical to maintain the information from which genomic DNA strand an RNA molecule was originally transcribed. This remains technically challenging (120), especially if one would like to maintain both strand specificity and full-length transcript coverage at the same time. As described above, template switching and IVT (Figure 2A and B) theoretically maintain strand specificity and full-length coverage. Yet the currently popular sequencing technologies only generate short reads. Moreover, current library preparation protocols require fragmentation of either the input RNA or the resulting cDNA. For bulk RNA-seq experiments, fragmentation of the RNA (120) prior to adapter ligation can rescue information about strand orientation. Due to the minute RNA amounts per cell, however, the currently used single-cell RNA-seq protocols consider fragmentation only after transcript amplification (i.e. on the cDNA level), meaning that strand specificity is lost. However, directional information can be preserved by compromising the full-length coverage and selecting either 5′ ends by affinity purification, STRT (*S*ingle-cell *T*agged *R*everse *T*ranscription) (37,112,121) (Figure 3A and Table 1) or 3′ ends by selective PCR after transcript fragmentation (113) (Figure 3B and Table 1).

### Cellular and molecular barcoding strategies

An in-depth single-cell RNA-seq analysis of a whole tissue may require the profiling of several thousands if not millions of representative individual cells. To reduce sequencing costs and increase throughput, previously developed methods that focus on just the 3′ or 5′ ends of transcripts have been modified for massively parallel RNA-seq of single cells (Figure 3) (112–113,121). The incorporation of a unique cellular identifier composed of a 4–5 bp random sequence in the template-switching oligonucleotide (Figure 3A) or in the oligo(dT) primer (Figure 3B) has made it possible to pool up to 1500 cells from a spleen for simultaneous sequencing (114); through the unique cellular barcode, each read could subsequently be assigned to its original cell.

Barcoding strategies can also be used to perform absolute quantification of each transcript in a single cell. Usually in RNA-seq, transcript abundance is quantified as RPKM and FPKM (reads/fragments per kilobase per million mapped reads) but these represent an indirect way to quantify RNA molecules and can be distorted by differential amplification efficiencies for different fragments. The absolute number of each transcript can be measured by barcoding every cDNA before amplification by inserting a unique random sequence at the 5′ end in the template switching oligonucleotide (37,122) or at the 3′ end in the oligo(dT) primer (114,115), respectively (Figure 3). Transcript quantification and data normalization based on ‘molecular counting’ compared to quantification based on the number of sequencing reads has been shown to improve reproducibility between different cells (37,115), especially for low abundant transcripts (<10 molecules/cell).

### Sub-single-cell sequencing: localizing transcripts within a cell

All the methods described above fail to preserve spatial information about the transcript inside the cell because the latter is lysed prior to detection. However, the localization of mRNAs within sub-cellular compartments is a cellular strategy to regulate gene expression (123), so it would be insightful to sequence cellular transcripts while preserving their natural context.

A first strategy consists of isolating a compartment of a cell and applying the previously described RNA-seq techniques. This has been achieved for single nuclei (124) as well as dendrites from neurons (125). Another strategy consists of sequencing RNA directly inside a cell without lysis (126,127), a method called ‘*in situ* sequencing’. Transcripts are converted into cDNA using gene-specific (126) or random primers (127), followed by rolling circle amplification and *in situ* sequencing-by-ligation using a fluorescence microscope. To fix their location inside the cell of interest, the transcripts are chemically linked to the protein matrix (127). The method has been successfully applied to fibroblasts (127) but has the potential to be applied to tissue sections and embryos. In particular, tissue and organ development and disease progression would greatly benefit from the preservation of the spatial context of individual cells. Proof-of-concept for such an *in situ* sequencing approach of tissue samples has already been described for a set of pre-selected genes (126), but also seems within reach on the genome-scale (127). The coupling of *in situ* sequencing and tissue-array technologies (128) could be envisaged to eventually enable the interrogation of hundreds of tissue samples in parallel.

## APPLICATIONS OF SINGLE-CELL RNA-SEQ

Once a cDNA library is prepared and sequenced, how does one interpret the data and make sense of cell-to-cell variability? In the next section, we will review cell-to-cell gene expression variability in two different contexts: first, when individual cells have the same genetic background (monoclonal cells) and, second, when stable genetic variants give rise to changes in gene expression among cells. Subsequently, we will detail single-cell RNA-seq applications in different areas of basic research and highlight medical implications.

### Similar but not the same

#### Same genetic background but different gene expression

The transcriptome of genetically identical cells within a population will differ on the single-cell level. Such cell-to-cell variability manifests itself in several different views of gene expression. A first level is global cell-to-cell expression variability. For example, when freshly isolated dendritic cells were compared by single-cell RNA-seq, the authors calculated a Pearson coefficient of only 0.48 in gene expression (43), which is in stark contrast to the 0.98 correlation observed when sequencing different populations each containing 10^5^ cells. An independent assessment of the observed cell-to-cell variability using an amplification-free method, i.e. image-based expression quantification with RNA FISH (43), validated the variability to reflect real biological differences in gene expression, at least for the highly expressed genes that were analyzed. Note that single-cell qRT-PCR is another commonly used method to independently validate cell-to-cell variations in gene expression. The second level of analysis is to compare the expression level of individual transcripts between different single cells and to plot their expression distribution. Analyzing immune cells after exposure to a bacterial antigen (43), it was found that housekeeping genes followed a log-normal distribution. Some other genes showed bimodal gene expression, meaning that these latter transcripts were lowly expressed in some cells and highly (at least one order of magnitude higher) in other cells (43). The last level of cell-to-cell variability considers splice variants. For instance, using SMART-seq to primarily analyze full-length transcripts (see above and Table 1), extensive variability in isoform variants was observed (43).

How can variability between cells with the same genetic content be explained? From a molecular point of view, single-cell RNA-seq confirms the previously observed widespread nature of stochastic gene expression. As reviewed elsewhere (38,129–130), a random assembly of RNA polymerase factors at the promoter influences the initial decision of whether and how efficiently a given gene will be transcribed. Single-cell RNA-seq has discovered novel facets of stochastic gene expression, for example, that stochasticity in allele-specific gene expression can affect up to one fourth of all somatic genes in embryonic and differentiated cells (44). While the underlying molecular mechanisms and functional consequences remain to be explained, one may speculate that such allele-specific variability could enable a small sub-set of cells that happen to have the ‘ideal’ gene expression program at the right time to rapidly adapt to external perturbations, thereby benefitting the entire population.

#### Different genetic backgrounds induce differential gene expression

While genetic mutations that impair the expression of an essential gene will be counter-selected, mutations that result in only slight or conditional expression changes will usually co-exist together with the wild-type cells in a population. These latter genetic variations produce a reservoir of genetically different cells and different transcriptomes.

Gene expression variation derived from genetic variants is referred to as expression quantitative trait loci (eQTLs). It has been analyzed in many studies to better understand disease-related pathways (131) but until recently, only on a population level. A systematic study of 92 genes concentrated on the Wnt signaling pathway from 15 individuals using high-throughput single-cell qRT-PCR (132,133) uncovered novel facets of eQTLs by linking single nucleotide polymorphisms (SNPs) to stochastic gene expression properties such as burst frequency and amplitude. We expect that this initial study will prompt genome-scale studies using single-cell RNA-seq and in-depth functional characterization of such genetic variants.

#### Data analysis framework

What are the functional implications of cell-to-cell variability? First, principal component analysis of single-cell RNA-seq data can reveal biologically distinct sub-populations, for example, ones that correspond to different developmental stages (43,114,134–137). Even closely related cells that apparently share the same phenotype could be discriminated, which is important to distinguish functionally different subtypes. Second, once cells are classified into distinct cell identities, genes can be clustered using co-variation analysis to extract regulatory circuits (43,45,51,137). For example, such co-expression analysis has revealed preferred signaling pathways among the different cell lineages of lung epithelial cells (137). Third, single-cell RNA-seq can dissect the temporal choreography of gene expression that underlies many processes of cell differentiation or reprogramming, as well as cellular responses to external stimuli (45,137). To facilitate this type of analysis, an unsupervised algorithm called Monocle (which does not rely upon known markers) has been introduced to unravel transcriptome dynamics (45). Importantly, the novel pathways identified could not have been revealed by RNA-seq of cell populations.

### Application of single-cell studies to basic research

The diverse applications of single-cell transcriptomics discussed below illustrate the power of the technique for redefining cell identities based on a molecular profiling and for the discovery of new cell differentiation routes. Single-cell transcriptomics has also been successfully applied to neurobiology, as reviewed in depth elsewhere (138).

#### Stem cell differentiation

Since stem cell differentiation is per se a single-cell decision process, studying the molecular basis of how stem cells commit to the differentiation process ultimately requires single-cell techniques. Single-cell RNA-seq has been used to dissect the development of the murine lung (137), identifying previously unknown lineage-specific markers of the different cell subtypes that constitute this organ. Another study (45) that aimed to resolve the differentiation trajectory of skeletal muscles subjected human primary myoblasts to single-cell RNA-seq at different time points during differentiation *in vitro*. This study identified eight transcription factors required to direct the differential expression of >1000 genes during the individual phases of differentiation.

#### Embryogenesis

Embryonic development can be considered as the differentiation transition from the cellular to the whole-organism level. Studying the early stages of embryonic development demands methods that are compatible with minute quantities of cells. Single-cell RNA-seq studies have enabled a global analysis of early mammalian development (93,134,139–141), helping to substitute hypothesis- with discovery-driven science. All these studies relied on the poly(A) tailing protocol (Figure 2A), thus focusing on mRNA expression. New insights into early embryogenesis include, for instance, major changes in mRNA isoform abundance and defined patterns of allele-specific gene expression during murine blastomere development (93,142), functional modules of co-regulated genes (141), and the first lncRNA expression maps of embryonic stem cells (ESCs) and human preimplantation embryos (140). We expect that RNA-seq will also improve single-cell analyses of sub-regions of the embryo, for example, the inner cell mass of murine blastocysts from which individual cells have already been analyzed via qRT-PCR and microarray (143).

#### Whole-tissue analysis

Dissecting the transcriptome of all the cells from a tissue will provide an opportunity to redefine our knowledge of lineage hierarchy with unprecedented molecular resolution. Massive parallel single-cell RNA-seq (114) of thousands of cells from the spleen without prior selection based on an *a priori* cell-surface marker combined with unsupervised hierarchical clustering was used to reconstitute the global cell heterogeneity within splenic tissue. Notably, the cells could be grouped into seven large sub-populations and dendritic cells could be further sub-classified into four groups. Upon exposure to a bacterial antigen, this single-cell RNA-seq method (114) revealed the reorganization of cell sub-populations within the tissue and show the emergence of new sub-populations with potentially functional roles that have not yet been characterized. Finally, this leads to the question if single-cell techniques can be extended to study the transcriptome of a whole organism?

#### Single-cell RNA-seq for whole-organism studies

A major goal in studying embryogenesis and organogenesis is to understand how single cells divide and differentiate to eventually build up an entire organism (1). There is a comprehensive knowledge of lineage commitment of every single cell in the body of the model worm *Caenorhabditis elegans* (144). Consequently, this organism was selected as a model system to establish the CEL-seq technique (Figure 2B and Table 1) (113). Profiling of the early stages of embryonic development in *C. elegans* discovered, for example, extensive transcriptional activity at developmental stages that had previously been considered transcriptionally inert (113). Similar to unrelated findings in mouse blastocysts (143), heterogeneity in the transcriptomes of individual cells seems to be a prerequisite for them to segregate into different lineages.

### A medical perspective on single-cell studies

Considering the rapid development of sequencing methods, one can expect single-cell RNA-seq to soon enter the clinics to facilitate more personalized therapeutic decisions for patients. In addition, the analysis of minimal invasive samples (from blood or fine-needle aspirates) instead of whole tissue holds the promise of enabling a rapid point-of-care diagnostic (145). Over a decade ago, individual cells that had disseminated from a primary tumor to the bone marrow in chemotherapy patients were analyzed (146). Whole-transcriptome amplification followed by microarray analysis suggested a now-established link between the integral plasma membrane protein CD147/EMMPRIN and tumor invasiveness and chemoresistance (147). Aberrant expression of several membrane proteins was also observed in the first RNA-seq study of single CTCs isolated from peripheral blood of melanoma patients (108). These proteins are thought to contribute to the invasiveness of CTCs and their ability to escape the immune system. Using the SMART-seq protocol, the authors captured almost the full-length transcripts (Figure 2A and Table 1), and so could look for SNPs that identified CTCs derived from melanoma. There is great hope that RNA-seq of CTCs will aid the identification of a tumor's origin, improving the treatment of patients.

## FUTURE CHALLENGES OF SINGLE-CELL RNA-SEQ

While the focus of single-cell RNA-seq has thus far been on polyadenylated mRNAs of eukaryotes, many other transcript classes remain to be fully explored. Moreover, modern biology explores a huge range of organisms, including many infectious prokaryotes, studies of which will require further development of the current methods for single-cell transcriptomics to be attempted. Below we will discuss the limitations that need to be overcome to reap the maximum benefits from single-cell RNA-seq.

Single-cell RNA-seq still requires significant further development before it provides a comprehensive view of the complete transcriptome of any given cell. Individual improvements notwithstanding, the current approaches suffer from a number of problems. For example, it is difficult to maintain strand specificity *and* detect isoform variants at the same time (Figure 3) when short read-based sequencing is used. RNA losses are estimated to be between 50 and 60% (37,44), with a much higher risk for low abundant transcripts (<5–10 transcripts per cell). Non-polyadenylated RNA species, in spite of them being the major fraction of transcripts in many important organisms, are currently under-represented, and post-transcriptional RNA modifications (148,149) and RNA editing events (150) have not at all been explored in single cells.

Some of these problems are being remedied. Long-read sequencing technologies have enabled RNA-seq with a median read length of up to 1.5 kb (101,151). Adapting these methods to single cells will abolish the compromise between strand specificity and full-length coverage. To capture poly(A)− and poly(A)+ transcripts simultaneously, several strategies are currently being developed. These include the use of ‘not-so-random’ primers that are bioinformatically predicted to bind to all cellular transcripts except ribosomal RNA (152). In addition, 5′-monophosphate-dependent exonuclease treatment for the removal of abundant stable RNAs has been optimized for small input amounts (97).

The limited sensitivity of single-cell RNA-seq is another limiting factor at the moment. It remains currently difficult to distinguish between technical noise and biological variability for low-abundance (∽10 copies/cell) transcripts (37,115), resulting in a considerable loss of information from cellular transcriptomes (43). For example, lncRNAs, even though typically present in only few copies per cell, can have important regulatory functions (153). Thus, sensitivity needs to be dramatically improved such that even transcripts with a single copy per cell can be quantitatively detected to fully understand such regulatory processes at the single-cell level.

Other important transcript classes that have been neglected in previous single-cell RNA-seq studies are microRNAs and other small RNAs with a length of less than 30 nucleotides. Multiplexed qRT-PCR has been used to analyze 220 microRNA in single ESCs (154,155), but in order to profile all of the predicted 2500 different human microRNAs (156), RNA-seq again will be the method of choice. In fact, this should already be possible with the current protocols.

Today's single-cell studies are typically conducted with dissociated cells. Since RNA-seq can be applied to an indefinite number of cells (114), at some point this may enable researchers to assemble the transcriptome of a whole organ. However, maintaining the 3D information of tissue architecture at the same time as sequencing remains a challenge. Laser microdissection would be too laborious for an entire organ and the method itself is not appropriate for complex tissues such as the brain. One could imagine combining lineage tracking methods with multicolor-imaging (157) and single-cell RNA-seq. For example, the already discussed *in situ* sequencing (127) can detect thousands of transcripts while maintaining the natural environment of the cell either from cells grown in a monolayer or a tissue section. Finally, the general lack of poly(A) tails has clearly hampered progress in single-cell transcriptomics of prokaryotic cells (97,158). However, we predict this will become increasingly important in the coming years because more than 99% of prokaryotes cannot be cultivated (159). Metatranscriptomics has already been applied on the population level and revealed the existence of new small RNAs (160). Given their importance for many infectious diseases as well as biotechnological applications, bacteria lend themselves for RNA-seq studies of decision-making processes in single cells.

**Figure 4.**
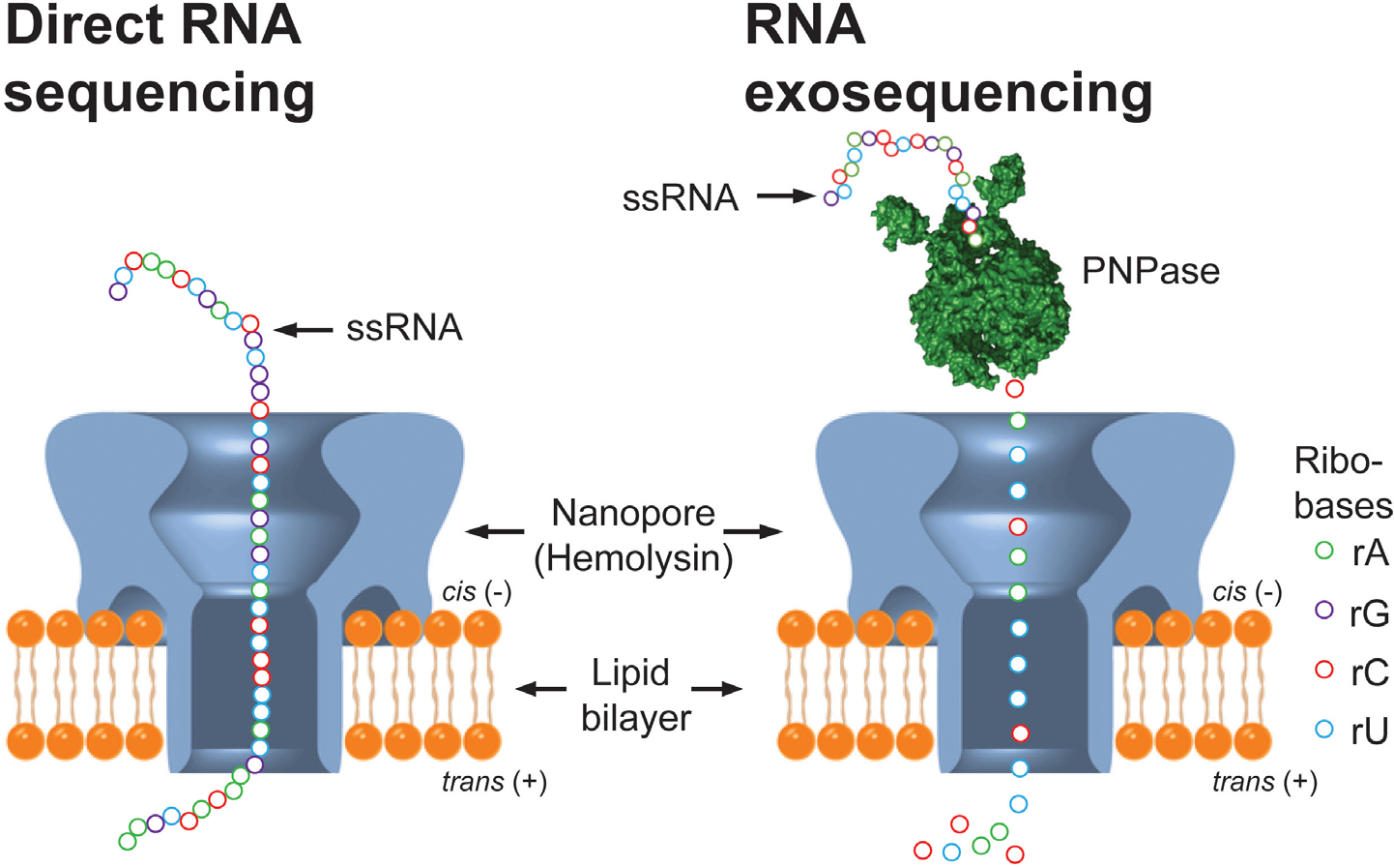
Envisioned strategies for nanopore-based RNA-seq. Left: while a single-stranded RNA stretch is translocated through a hemolysin pore in a membrane, each ribobase could be detected by measuring current changes between the *cis* (−) and *trans* (+) compartment divided by the lipid bilayer. Right: single-stranded RNA is cleaved by polynucleotide phosphorylase (PNPase) and each released ribobase could be read separately by measuring the current between the *cis* (−) and *trans* (+) compartment. Nanopore drawing is reproduced from (169) by permission from Macmillan Publishers Ltd on behalf of Scientific Reports, copyright 2013. RNA exosequencing principle is adapted with permission from (167) and copyright 2013 American Chemical Society.

Many of the scientific challenges discussed above might be solved with the anticipated next wave in the sequencing revolution via nanopores. Briefly, in nanopore-based sequencing the identity and sequential order of nucleotides is determined as the nucleic acid molecule of interest is threaded through one of many tiny (nano)pores in a membrane (161–163,168). Of note, the nanopore principle theoretically holds the promise of direct RNA-sequencing (i.e. without a cDNA intermediate) without size discrimination, down to the single-molecule level and very low cost. Although no hard data are yet available, it is claimed that the nanopore sequencers under development can already sequence single-stranded DNA of up to few kilobases (164). For direct RNA-sequencing via the nanopore principle, two strategies have been envisioned. First, as outlined above each nucleotide can be read while the whole RNA molecule is translocated through the pore (Figure 4, left part). It has been demonstrated that, as they cross the pore, individual ribonucleotides can be distinguished based on ionic current flow changes (165). Moreover, RNA strands as long as 6 kb can be threaded through the pore (166), but interestingly RNA translocation is currently too fast to allow accurate reading of ‘one nucleotide after the other’ and therefore molecules that slow down translocation are being introduced. The other strategy is based on RNA exosequencing (167) (Figure 4, right part), i.e. RNA is successively cleaved by polynucleotide phosphorylase (PNPase) (168) and each released nucleotide is read separately in the nanopore. Either way, as direct RNA-sequencing would render both reverse transcription and amplification obsolete, it appears to be the ultimate gold standard for single-cell transcriptomics.

## CONCLUSION

RNA-seq has revolutionized transcriptomics and rapidly become the method of choice to address both quantitative and qualitative aspects of gene expression. Nonetheless, most studies have analyzed the average transcriptome of a whole population of cells. The recent years, however, have taught us that many important cellular aspects can only be assessed with the help of single-cell approaches. Examples include mono-allelic gene expression, lineage tracing during cellular differentiation and organ or embryo development in eukaryotes, as well as bi-stable gene expression, biofilm formation or persister cell formation in bacteria. A major future challenge will be to go beyond the poly(A) transcriptome of eukaryotes and bring single-cell RNA-seq to the level that *all* types of cellular transcripts are analyzed in parallel. Important first steps toward single-bacterium transcriptomics have been taken (97). As cDNA synthesis dictates the transcript classes to be captured and represents the material-limiting and most length bias-prone step in the experimental pipeline, the long-term goal must be to directly sequence full-length RNA molecules. With such powerful techniques researchers could eventually address ambitious projects such as global expression maps of low-abundance lncRNAs in single mammalian cells or a new type of Dual RNA-seq of infected single cells in which all eukaryotic transcripts of the host are read simultaneously with those from an intracellular bacterial pathogen (170).

## References

[1] Shapiro E., Biezuner T., Linnarsson S. (2013). Single-cell sequencing-based technologies will revolutionize whole-organism science. Nat. Rev. Genet..

[2] Snijder B., Sacher R., Ramo P., Damm E.M., Liberali P., Pelkmans L. (2009). Population context determines cell-to-cell variability in endocytosis and virus infection. Nature.

[3] Sharma S.V., Lee D.Y., Li B., Quinlan M.P., Takahashi F., Maheswaran S., McDermott U., Azizian N., Zou L., Fischbach M.A. (2010). A chromatin-mediated reversible drug-tolerant state in cancer cell subpopulations. Cell.

[4] Helaine S., Cheverton A.M., Watson K.G., Faure L.M., Matthews S.A., Holden D.W. (2014). Internalization of Salmonella by macrophages induces formation of nonreplicating persisters. Science.

[5] Baccelli I., Schneeweiss A., Riethdorf S., Stenzinger A., Schillert A., Vogel V., Klein C., Saini M., Bauerle T., Wallwiener M. (2013). Identification of a population of blood circulating tumor cells from breast cancer patients that initiates metastasis in a xenograft assay. Nat. Biotechnol..

[6] Rinke C., Schwientek P., Sczyrba A., Ivanova N.N., Anderson I.J., Cheng J.F., Darling A., Malfatti S., Swan B.K., Gies E.A. (2013). Insights into the phylogeny and coding potential of microbial dark matter. Nature.

[7] Metzker M.L. (2010). Sequencing technologies—the next generation. Nat. Rev. Genet..

[8] Bendall S.C., Nolan G.P. (2012). From single cells to deep phenotypes in cancer. Nat. Biotechnol..

[9] Kalisky T., Blainey P., Quake S.R. (2011). Genomic analysis at the single-cell level. Annu. Rev. Genet..

[10] Nagalakshmi U., Wang Z., Waern K., Shou C., Raha D., Gerstein M., Snyder M. (2008). The transcriptional landscape of the yeast genome defined by RNA sequencing. Science.

[11] Wang Z., Gerstein M., Snyder M. (2009). RNA-Seq: a revolutionary tool for transcriptomics. Nat. Rev. Genet..

[12] Djebali S., Davis C.A., Merkel A., Dobin A., Lassmann T., Mortazavi A., Tanzer A., Lagarde J., Lin W., Schlesinger F. (2012). Landscape of transcription in human cells. Nature.

[13] Hangauer M.J., Vaughn I.W., McManus M.T. (2013). Pervasive transcription of the human genome produces thousands of previously unidentified long intergenic noncoding RNAs. PLoS Genet..

[14] Pelechano V., Wei W., Steinmetz L.M. (2013). Extensive transcriptional heterogeneity revealed by isoform profiling. Nature.

[15] Vogel J., Bartels V., Tang T.H., Churakov G., Slagter-Jager J.G., Huttenhofer A., Wagner E.G. (2003). RNomics in Escherichia coli detects new sRNA species and indicates parallel transcriptional output in bacteria. Nucleic Acids Res..

[16] Kroger C., Dillon S.C., Cameron A.D., Papenfort K., Sivasankaran S.K., Hokamp K., Chao Y., Sittka A., Hebrard M., Handler K. (2012). The transcriptional landscape and small RNAs of Salmonella enterica serovar Typhimurium. Proc. Natl. Acad. Sci. U.S.A..

[17] Albrecht M., Sharma C.M., Dittrich M.T., Muller T., Reinhardt R., Vogel J., Rudel T. (2011). The transcriptional landscape of Chlamydia pneumoniae. Genome Biol..

[18] Nicolas P., Mader U., Dervyn E., Rochat T., Leduc A., Pigeonneau N., Bidnenko E., Marchadier E., Hoebeke M., Aymerich S. (2012). Condition-dependent transcriptome reveals high-level regulatory architecture in Bacillus subtilis. Science.

[19] Irnov I., Sharma C.M., Vogel J., Winkler W.C. (2010). Identification of regulatory RNAs in Bacillus subtilis. Nucleic Acids Res..

[20] Guell M., van Noort V., Yus E., Chen W.H., Leigh-Bell J., Michalodimitrakis K., Yamada T., Arumugam M., Doerks T., Kuhner S. (2009). Transcriptome complexity in a genome-reduced bacterium. Science.

[21] Sharma C.M., Hoffmann S., Darfeuille F., Reignier J., Findeiss S., Sittka A., Chabas S., Reiche K., Hackermuller J., Reinhardt R. (2010). The primary transcriptome of the major human pathogen Helicobacter pylori. Nature.

[22] Lasa I., Toledo-Arana A., Dobin A., Villanueva M., de los Mozos I.R., Vergara-Irigaray M., Segura V., Fagegaltier D., Penades J.R., Valle J. (2011). Genome-wide antisense transcription drives mRNA processing in bacteria. Proc. Natl. Acad. Sci. U.S.A..

[23] Mraheil M.A., Billion A., Mohamed W., Mukherjee K., Kuenne C., Pischimarov J., Krawitz C., Retey J., Hartsch T., Chakraborty T. (2011). The intracellular sRNA transcriptome of Listeria monocytogenes during growth in macrophages. Nucleic Acids Res..

[24] Wurtzel O., Sesto N., Mellin J.R., Karunker I., Edelheit S., Becavin C., Archambaud C., Cossart P., Sorek R. (2012). Comparative transcriptomics of pathogenic and non-pathogenic Listeria species. Mol. Syst. Biol..

[25] Dugar G., Herbig A., Forstner K.U., Heidrich N., Reinhardt R., Nieselt K., Sharma C.M. (2013). High-resolution transcriptome maps reveal strain-specific regulatory features of multiple Campylobacter jejuni isolates. PLoS Genet..

[26] Georg J., Hess W.R. (2011). cis-Antisense RNA, another level of gene regulation in bacteria. Microbiol. Mol. Biol. Rev..

[27] Wurtzel O., Sapra R., Chen F., Zhu Y., Simmons B.A., Sorek R. (2010). A single-base resolution map of an archaeal transcriptome. Genome Res..

[28] Kapranov P., Willingham A.T., Gingeras T.R. (2007). Genome-wide transcription and the implications for genomic organization. Nat. Rev. Genet..

[29] Kapranov P., Cheng J., Dike S., Nix D.A., Duttagupta R., Willingham A.T., Stadler P.F., Hertel J., Hackermuller J., Hofacker I.L. (2007). RNA maps reveal new RNA classes and a possible function for pervasive transcription. Science.

[30] Pelechano V., Steinmetz L.M. (2013). Gene regulation by antisense transcription. Nat. Rev. Genet..

[31] Jacquier A. (2009). The complex eukaryotic transcriptome: unexpected pervasive transcription and novel small RNAs. Nat. Rev. Genet..

[32] Rouskin S., Zubradt M., Washietl S., Kellis M., Weissman J.S. (2014). Genome-wide probing of RNA structure reveals active unfolding of mRNA structures in vivo. Nature.

[33] Park E., Williams B., Wold B.J., Mortazavi A. (2012). RNA editing in the human ENCODE RNA-seq data. Genome Res..

[34] Geisberg J.V., Moqtaderi Z., Fan X., Ozsolak F., Struhl K. (2014). Global analysis of mRNA isoform half-lives reveals stabilizing and destabilizing elements in yeast. Cell.

[35] Ingolia N.T., Ghaemmaghami S., Newman J.R., Weissman J.S. (2009). Genome-wide analysis in vivo of translation with nucleotide resolution using ribosome profiling. Science.

[36] Castello A., Fischer B., Eichelbaum K., Horos R., Beckmann B.M., Strein C., Davey N.E., Humphreys D.T., Preiss T., Steinmetz L.M. (2012). Insights into RNA biology from an atlas of mammalian mRNA-binding proteins. Cell.

[37] Islam S., Zeisel A., Joost S., La Manno G., Zajac P., Kasper M., Lonnerberg P., Linnarsson S. (2014). Quantitative single-cell RNA-seq with unique molecular identifiers. Nat. Methods.

[38] Sanchez A., Golding I. (2013). Genetic determinants and cellular constraints in noisy gene expression. Science.

[39] Marguerat S., Schmidt A., Codlin S., Chen W., Aebersold R., Bahler J. (2012). Quantitative analysis of fission yeast transcriptomes and proteomes in proliferating and quiescent cells. Cell.

[40] Taniguchi Y., Choi P.J., Li G.W., Chen H., Babu M., Hearn J., Emili A., Xie X.S. (2010). Quantifying E. coli proteome and transcriptome with single-molecule sensitivity in single cells. Science.

[41] Chang H.H., Hemberg M., Barahona M., Ingber D.E., Huang S. (2008). Transcriptome-wide noise controls lineage choice in mammalian progenitor cells. Nature.

[42] Levsky J.M., Shenoy S.M., Pezo R.C., Singer R.H. (2002). Single-cell gene expression profiling. Science.

[43] Shalek A.K., Satija R., Adiconis X., Gertner R.S., Gaublomme J.T., Raychowdhury R., Schwartz S., Yosef N., Malboeuf C., Lu D. (2013). Single-cell transcriptomics reveals bimodality in expression and splicing in immune cells. Nature.

[44] Deng Q., Ramskold D., Reinius B., Sandberg R. (2014). Single-cell RNA-seq reveals dynamic, random monoallelic gene expression in mammalian cells. Science.

[45] Trapnell C., Cacchiarelli D., Grimsby J., Pokharel P., Li S., Morse M., Lennon N.J., Livak K.J., Mikkelsen T.S., Rinn J.L. (2014). The dynamics and regulators of cell fate decisions are revealed by pseudotemporal ordering of single cells. Nat. Biotechnol..

[46] Chattopadhyay P.K., Price D.A., Harper T.F., Betts M.R., Yu J., Gostick E., Perfetto S.P., Goepfert P., Koup R.A., De Rosa S.C. (2006). Quantum dot semiconductor nanocrystals for immunophenotyping by polychromatic flow cytometry. Nat. Med..

[47] Chattopadhyay P.K., Roederer M. (2012). Cytometry: today's technology and tomorrow's horizons. Methods.

[48] Gattinoni L., Lugli E., Ji Y., Pos Z., Paulos C.M., Quigley M.F., Almeida J.R., Gostick E., Yu Z., Carpenito C. (2011). A human memory T cell subset with stem cell-like properties. Nat. Med..

[49] Dalerba P., Kalisky T., Sahoo D., Rajendran P.S., Rothenberg M.E., Leyrat A.A., Sim S., Okamoto J., Johnston D.M., Qian D. (2011). Single-cell dissection of transcriptional heterogeneity in human colon tumors. Nat. Biotechnol..

[50] Irish J.M., Hovland R., Krutzik P.O., Perez O.D., Bruserud O., Gjertsen B.T., Nolan G.P. (2004). Single cell profiling of potentiated phospho-protein networks in cancer cells. Cell.

[51] Janes K.A., Wang C.C., Holmberg K.J., Cabral K., Brugge J.S. (2010). Identifying single-cell molecular programs by stochastic profiling. Nat. Methods.

[52] Klemm S., Semrau S., Wiebrands K., Mooijman D., Faddah D.A., Jaenisch R., van Oudenaarden A. (2014). Transcriptional profiling of cells sorted by RNA abundance. Nat. Methods.

[53] Ponten F., Gry M., Fagerberg L., Lundberg E., Asplund A., Berglund L., Oksvold P., Bjorling E., Hober S., Kampf C. (2009). A global view of protein expression in human cells, tissues, and organs. Mol. Syst. Biol..

[54] Saliba A.E., Saias L., Psychari E., Minc N., Simon D., Bidard F.C., Mathiot C., Pierga J.Y., Fraisier V., Salamero J. (2010). Microfluidic sorting and multimodal typing of cancer cells in self-assembled magnetic arrays. Proc. Natl. Acad. Sci. U.S.A..

[55] Filby A., Perucha E., Summers H., Rees P., Chana P., Heck S., Lord G.M., Davies D. (2011). An imaging flow cytometric method for measuring cell division history and molecular symmetry during mitosis. Cytometry A.

[56] Goda K., Ayazi A., Gossett D.R., Sadasivam J., Lonappan C.K., Sollier E., Fard A.M., Hur S.C., Adam J., Murray C. (2012). High-throughput single-microparticle imaging flow analyzer. Proc. Natl. Acad. Sci. U.S.A..

[57] Morris J., Singh J.M., Eberwine J.H. (2011). Transcriptome analysis of single cells. J. Vis. Exp..

[58] Citri A., Pang Z.P., Sudhof T.C., Wernig M., Malenka R.C. (2011). Comprehensive qPCR profiling of gene expression in single neuronal cells. Nat. Protoc..

[59] Nagy A., Gertsensten M., Vintersten K., Behringer R. (2003). Manipulating the Mouse Embryo: A Laboratory Manual.

[60] Frohlich J., Konig H. (2000). New techniques for isolation of single prokaryotic cells. FEMS Microbiol. Rev..

[61] Huber R., Burggraf S., Mayer T., Barns S.M., Rossnagel P., Stetter K.O. (1995). Isolation of a hyperthermophilic archaeum predicted by in situ RNA analysis. Nature.

[62] Yoshimoto N., Kida A., Jie X., Kurokawa M., Iijima M., Niimi T., Maturana A.D., Nikaido I., Ueda H.R., Tatematsu K. (2013). An automated system for high-throughput single cell-based breeding. Sci. Rep..

[63] Kovac J.R., Voldman J. (2007). Intuitive, image-based cell sorting using optofluidic cell sorting. Anal. Chem..

[64] Blainey P.C., Mosier A.C., Potanina A., Francis C.A., Quake S.R. (2011). Genome of a low-salinity ammonia-oxidizing archaeon determined by single-cell and metagenomic analysis. PLoS One.

[65] Landry Z.C., Giovanonni S.J., Quake S.R., Blainey P.C. (2013). Optofluidic cell selection from complex microbial communities for single-genome analysis. Methods Enzymol..

[66] Sackmann E.K., Fulton A.L., Beebe D.J. (2014). The present and future role of microfluidics in biomedical research. Nature.

[67] Marcy Y., Ouverney C., Bik E.M., Losekann T., Ivanova N., Martin H.G., Szeto E., Platt D., Hugenholtz P., Relman D.A. (2007). Dissecting biological "dark matter" with single-cell genetic analysis of rare and uncultivated TM7 microbes from the human mouth. Proc. Natl. Acad. Sci. U.S.A..

[68] Blainey P.C., Quake S.R. (2011). Digital MDA for enumeration of total nucleic acid contamination. Nucleic Acids Res..

[69] Davis J.A., Inglis D.W., Morton K.J., Lawrence D.A., Huang L.R., Chou S.Y., Sturm J.C., Austin R.H. (2006). Deterministic hydrodynamics: taking blood apart. Proc. Natl. Acad. Sci. U.S.A..

[70] Gossett D.R., Tse H.T., Lee S.A., Ying Y., Lindgren A.G., Yang O.O., Rao J., Clark A.T., Di Carlo D. (2012). Hydrodynamic stretching of single cells for large population mechanical phenotyping. Proc. Natl. Acad. Sci. U.S.A..

[71] Zhang W., Kai K., Choi D.S., Iwamoto T., Nguyen Y.H., Wong H., Landis M.D., Ueno N.T., Chang J., Qin L. (2012). Microfluidics separation reveals the stem-cell-like deformability of tumor-initiating cells. Proc. Natl. Acad. Sci. U.S.A..

[72] Vahey M.D., Quiros Pesudo L., Svensson J.P., Samson L.D., Voldman J. (2013). Microfluidic genome-wide profiling of intrinsic electrical properties in Saccharomyces cerevisiae. Lab Chip.

[73] Huang R., Barber T.A., Schmidt M.A., Tompkins R.G., Toner M., Bianchi D.W., Kapur R., Flejter W.L. (2008). A microfluidics approach for the isolation of nucleated red blood cells (NRBCs) from the peripheral blood of pregnant women. Prenat. Diagn..

[74] Tse H.T., Gossett D.R., Moon Y.S., Masaeli M., Sohsman M., Ying Y., Mislick K., Adams R.P., Rao J., Di Carlo D. (2013). Quantitative diagnosis of malignant pleural effusions by single-cell mechanophenotyping. Sci. Transl. Med..

[75] Karabacak N.M., Spuhler P.S., Fachin F., Lim E.J., Pai V., Ozkumur E., Martel J.M., Kojic N., Smith K., Chen P.I. (2014). Microfluidic, marker-free isolation of circulating tumor cells from blood samples. Nat. Protoc..

[76] Ozkumur E., Shah A.M., Ciciliano J.C., Emmink B.L., Miyamoto D.T., Brachtel E., Yu M., Chen P.I., Morgan B., Trautwein J. (2013). Inertial focusing for tumor antigen-dependent and -independent sorting of rare circulating tumor cells. Sci. Transl. Med..

[77] Yu M., Bardia A., Wittner B.S., Stott S.L., Smas M.E., Ting D.T., Isakoff S.J., Ciciliano J.C., Wells M.N., Shah A.M. (2013). Circulating breast tumor cells exhibit dynamic changes in epithelial and mesenchymal composition. Science.

[78] Yu M., Ting D.T., Stott S.L., Wittner B.S., Ozsolak F., Paul S., Ciciliano J.C., Smas M.E., Winokur D., Gilman A.J. (2012). RNA sequencing of pancreatic circulating tumour cells implicates WNT signalling in metastasis. Nature.

[79] Landry J.J., Pyl P.T., Rausch T., Zichner T., Tekkedil M.M., Stutz A.M., Jauch A., Aiyar R.S., Pau G., Delhomme N. (2013). The genomic and transcriptomic landscape of a HeLa cell line. G3 (Bethesda).

[80] Ghosh S., Spagnoli G.C., Martin I., Ploegert S., Demougin P., Heberer M., Reschner A. (2005). Three-dimensional culture of melanoma cells profoundly affects gene expression profile: a high density oligonucleotide array study. J. Cell. Physiol..

[81] Mammoto A., Mammoto T., Ingber D.E. (2012). Mechanosensitive mechanisms in transcriptional regulation. J. Cell. Sci..

[82] Luo L., Salunga R.C., Guo H., Bittner A., Joy K.C., Galindo J.E., Xiao H., Rogers K.E., Wan J.S., Jackson M.R. (1999). Gene expression profiles of laser-captured adjacent neuronal subtypes. Nat. Med..

[83] Wang L., Janes K.A. (2013). Stochastic profiling of transcriptional regulatory heterogeneities in tissues, tumors and cultured cells. Nat. Protoc..

[84] Espina V., Wulfkuhle J.D., Calvert V.S., VanMeter A., Zhou W., Coukos G., Geho D.H., Petricoin E.F., Liotta L.A. (2006). Laser-capture microdissection. Nat. Protoc..

[85] White A.K., VanInsberghe M., Petriv O.I., Hamidi M., Sikorski D., Marra M.A., Piret J., Aparicio S., Hansen C.L. (2011). High-throughput microfluidic single-cell RT-qPCR. Proc. Natl. Acad. Sci. U.S.A..

[86] White A.K., Heyries K.A., Doolin C., Vaninsberghe M., Hansen C.L. (2013). High-throughput microfluidic single-cell digital polymerase chain reaction. Anal. Chem..

[87] Livak K.J., Wills Q.F., Tipping A.J., Datta K., Mittal R., Goldson A.J., Sexton D.W., Holmes C.C. (2013). Methods for qPCR gene expression profiling applied to 1440 lymphoblastoid single cells. Methods.

[88] Hartmann C.H., Klein C.A. (2006). Gene expression profiling of single cells on large-scale oligonucleotide arrays. Nucleic Acids Res..

[89] Nygaard V., Hovig E. (2006). Options available for profiling small samples: a review of sample amplification technology when combined with microarray profiling. Nucleic Acids Res..

[90] Kurimoto K., Yabuta Y., Ohinata Y., Saitou M. (2007). Global single-cell cDNA amplification to provide a template for representative high-density oligonucleotide microarray analysis. Nat. Protoc..

[91] Picelli S., Bjorklund A.K., Faridani O.R., Sagasser S., Winberg G., Sandberg R. (2013). Smart-seq2 for sensitive full-length transcriptome profiling in single cells. Nat. Methods.

[92] Adey A., Morrison H.G., Asan, Xun X., Kitzman J.O., Turner E.H., Stackhouse B., MacKenzie A.P., Caruccio N.C., Zhang X. (2010). Rapid, low-input, low-bias construction of shotgun fragment libraries by high-density in vitro transposition. Genome Biol..

[93] Tang F., Barbacioru C., Wang Y., Nordman E., Lee C., Xu N., Wang X., Bodeau J., Tuch B.B., Siddiqui A. (2009). mRNA-Seq whole-transcriptome analysis of a single cell. Nat. Methods.

[94] He S., Wurtzel O., Singh K., Froula J.L., Yilmaz S., Tringe S.G., Wang Z., Chen F., Lindquist E.A., Sorek R. (2010). Validation of two ribosomal RNA removal methods for microbial metatranscriptomics. Nat. Methods.

[95] Yang L., Duff M.O., Graveley B.R., Carmichael G.G., Chen L.L. (2011). Genomewide characterization of non-polyadenylated RNAs. Genome Biol..

[96] Guell M., Yus E., Lluch-Senar M., Serrano L. (2011). Bacterial transcriptomics: what is beyond the RNA horiz-ome?. Nat. Rev. Microbiol..

[97] Kang Y., Norris M.H., Zarzycki-Siek J., Nierman W.C., Donachie S.P., Hoang T.T. (2011). Transcript amplification from single bacterium for transcriptome analysis. Genome Res..

[98] Gerard G.F., D'Alessio J.M., Kotewicz M.L., Noon M.C. (1986). Influence on stability in Escherichia coli of the carboxy-terminal structure of cloned Moloney murine leukemia virus reverse transcriptase. DNA.

[99] Arezi B., Hogrefe H. (2009). Novel mutations in Moloney Murine Leukemia Virus reverse transcriptase increase thermostability through tighter binding to template-primer. Nucleic Acids Res..

[100] Stahlberg A., Kubista M., Pfaffl M. (2004). Comparison of reverse transcriptases in gene expression analysis. Clin. Chem..

[101] Sharon D., Tilgner H., Grubert F., Snyder M. (2013). A single-molecule long-read survey of the human transcriptome. Nat. Biotechnol..

[102] Lazinski D.W., Camilli A. (2013). Homopolymer tail-mediated ligation PCR: a streamlined and highly efficient method for DNA cloning and library construction. Biotechniques.

[103] Belyavsky A., Vinogradova T., Rajewsky K. (1989). PCR-based cDNA library construction: general cDNA libraries at the level of a few cells. Nucleic Acids Res..

[104] Kurimoto K., Yabuta Y., Ohinata Y., Ono Y., Uno K.D., Yamada R.G., Ueda H.R., Saitou M. (2006). An improved single-cell cDNA amplification method for efficient high-density oligonucleotide microarray analysis. Nucleic Acids Res..

[105] Tang F., Barbacioru C., Nordman E., Li B., Xu N., Bashkirov V.I., Lao K., Surani M.A. (2010). RNA-Seq analysis to capture the transcriptome landscape of a single cell. Nat. Protoc..

[106] Sasagawa Y., Nikaido I., Hayashi T., Danno H., Uno K.D., Imai T., Ueda H.R. (2013). Quartz-Seq: a highly reproducible and sensitive single-cell RNA sequencing method, reveals non-genetic gene-expression heterogeneity. Genome Biol..

[107] Streets A.M., Zhang X., Cao C., Pang Y., Wu X., Xiong L., Yang L., Fu Y., Zhao L., Tang F. (2014). Microfluidic single-cell whole-transcriptome sequencing. Proc. Natl. Acad. Sci. U.S.A..

[108] Ramskold D., Luo S., Wang Y.C., Li R., Deng Q., Faridani O.R., Daniels G.A., Khrebtukova I., Loring J.F., Laurent L.C. (2012). Full-length mRNA-Seq from single-cell levels of RNA and individual circulating tumor cells. Nat. Biotechnol..

[109] Picelli S., Faridani O.R., Bjorklund A.K., Winberg G., Sagasser S., Sandberg R. (2014). Full-length RNA-seq from single cells using Smart-seq2. Nat. Protoc..

[110] Eberwine J., Yeh H., Miyashiro K., Cao Y., Nair S., Finnell R., Zettel M., Coleman P. (1992). Analysis of gene expression in single live neurons. Proc. Natl. Acad. Sci. U.S.A..

[111] Pan X., Durrett R.E., Zhu H., Tanaka Y., Li Y., Zi X., Marjani S.L., Euskirchen G., Ma C., Lamotte R.H. (2013). Two methods for full-length RNA sequencing for low quantities of cells and single cells. Proc. Natl. Acad. Sci. U.S.A..

[112] Islam S., Kjallquist U., Moliner A., Zajac P., Fan J.B., Lonnerberg P., Linnarsson S. (2012). Highly multiplexed and strand-specific single-cell RNA 5' end sequencing. Nat. Protoc..

[113] Hashimshony T., Wagner F., Sher N., Yanai I. (2012). CEL-Seq: single-cell RNA-Seq by multiplexed linear amplification. Cell Rep..

[114] Jaitin D.A., Kenigsberg E., Keren-Shaul H., Elefant N., Paul F., Zaretsky I., Mildner A., Cohen N., Jung S., Tanay A. (2014). Massively parallel single-cell RNA-seq for marker-free decomposition of tissues into cell types. Science.

[115] Grun D., Kester L., van Oudenaarden A. (2014). Validation of noise models for single-cell transcriptomics. Nat. Methods.

[116] Picelli S., Bjorklund A.K., Faridani O.R., Sagasser S., Winberg G., Sandberg R. (2013). Smart-seq2 for sensitive full-length transcriptome profiling in single cells. Nat. Methods.

[117] Zhu Y.Y., Machleder E.M., Chenchik A., Li R., Siebert P.D. (2001). Reverse transcriptase template switching: a SMART approach for full-length cDNA library construction. Biotechniques.

[118] Lovatt D., Ruble B.K., Lee J., Dueck H., Kim T.K., Fisher S., Francis C., Spaethling J.M., Wolf J.A., Grady M.S. (2014). Transcriptome in vivo analysis (TIVA) of spatially defined single cells in live tissue. Nat. Methods.

[119] Sambrook J., Russell D.W. (2006). Construction of cDNA libraries * stage 2: second-strand synthesis. CSH Protoc..

[120] Levin J.Z., Yassour M., Adiconis X., Nusbaum C., Thompson D.A., Friedman N., Gnirke A., Regev A. (2010). Comprehensive comparative analysis of strand-specific RNA sequencing methods. Nat. Methods.

[121] Islam S., Kjallquist U., Moliner A., Zajac P., Fan J.B., Lonnerberg P., Linnarsson S. (2011). Characterization of the single-cell transcriptional landscape by highly multiplex RNA-seq. Genome Res.

[122] Kivioja T., Vaharautio A., Karlsson K., Bonke M., Enge M., Linnarsson S., Taipale J. (2011). Counting absolute numbers of molecules using unique molecular identifiers. Nat. Methods.

[123] Martin K.C., Ephrussi A. (2009). mRNA localization: gene expression in the spatial dimension. Cell.

[124] Grindberg R.V., Yee-Greenbaum J.L., McConnell M.J., Novotny M., O'Shaughnessy A.L., Lambert G.M., Arauzo-Bravo M.J., Lee J., Fishman M., Robbins G.E. (2013). RNA-sequencing from single nuclei. Proc. Natl. Acad. Sci. U.S.A..

[125] Khaladkar M., Buckley P.T., Lee M.T., Francis C., Eghbal M.M., Chuong T., Suresh S., Kuhn B., Eberwine J., Kim J. (2013). Subcellular RNA sequencing reveals broad presence of cytoplasmic intron-sequence retaining transcripts in mouse and rat neurons. PLoS One.

[126] Ke R., Mignardi M., Pacureanu A., Svedlund J., Botling J., Wahlby C., Nilsson M. (2013). In situ sequencing for RNA analysis in preserved tissue and cells. Nat. Methods.

[127] Lee J.H., Daugharthy E.R., Scheiman J., Kalhor R., Yang J.L., Ferrante T.C., Terry R., Jeanty S.S., Li C., Amamoto R. (2014). Highly multiplexed subcellular RNA sequencing in situ. Science.

[128] Kononen J., Bubendorf L., Kallioniemi A., Barlund M., Schraml P., Leighton S., Torhorst J., Mihatsch M.J., Sauter G., Kallioniemi O.P. (1998). Tissue microarrays for high-throughput molecular profiling of tumor specimens. Nat. Med..

[129] Levine J.H., Lin Y., Elowitz M.B. (2013). Functional roles of pulsing in genetic circuits. Science.

[130] Coulon A., Chow C.C., Singer R.H., Larson D.R. (2013). Eukaryotic transcriptional dynamics: from single molecules to cell populations. Nat. Rev. Genet..

[131] Cookson W., Liang L., Abecasis G., Moffatt M., Lathrop M. (2009). Mapping complex disease traits with global gene expression. Nat. Rev. Genet..

[132] Wills Q.F., Livak K.J., Tipping A.J., Enver T., Goldson A.J., Sexton D.W., Holmes C. (2013). Single-cell gene expression analysis reveals genetic associations masked in whole-tissue experiments. Nat. Biotechnol..

[133] Petretto E. (2013). Single cell expression quantitative trait loci and complex traits. Genome Med..

[134] Tang F., Barbacioru C., Bao S., Lee C., Nordman E., Wang X., Lao K., Surani M.A. (2010). Tracing the derivation of embryonic stem cells from the inner cell mass by single-cell RNA-Seq analysis. Cell Stem Cell.

[135] Buganim Y., Faddah D.A., Cheng A.W., Itskovich E., Markoulaki S., Ganz K., Klemm S.L., van Oudenaarden A., Jaenisch R. (2012). Single-cell expression analyses during cellular reprogramming reveal an early stochastic and a late hierarchic phase. Cell.

[136] Trapnell C., Cacchiarelli D., Grimsby J., Pokharel P., Li S., Morse M., Lennon N.J., Livak K.J., Mikkelsen T.S., Rinn J.L. (2014). The dynamics and regulators of cell fate decisions are revealed by pseudotemporal ordering of single cells. Nat. Biotechnol.

[137] Treutlein B., Brownfield D.G., Wu A.R., Neff N., Mantalas G.L., Espinoza F.H., Desai T.J., Krasnow M.A., Quake S.R. (2014). Reconstructing lineage hierarchies of the distal lung epithelium using single-cell RNA-seq. Nature.

[138] Eberwine J., Lovatt D., Buckley P., Dueck H., Francis C., Kim T.K., Lee J., Lee M., Miyashiro K., Morris J. (2012). Quantitative biology of single neurons. J. R. Soc. Interface.

[139] Tang F., Barbacioru C., Nordman E., Bao S., Lee C., Wang X., Tuch B.B., Heard E., Lao K., Surani M.A. (2011). Deterministic and stochastic allele specific gene expression in single mouse blastomeres. PLoS One.

[140] Yan L., Yang M., Guo H., Yang L., Wu J., Li R., Liu P., Lian Y., Zheng X., Yan J. (2013). Single-cell RNA-Seq profiling of human preimplantation embryos and embryonic stem cells. Nat. Struct. Mol. Biol..

[141] Xue Z., Huang K., Cai C., Cai L., Jiang C.Y., Feng Y., Liu Z., Zeng Q., Cheng L., Sun Y.E. (2013). Genetic programs in human and mouse early embryos revealed by single-cell RNA sequencing. Nature.

[142] Tang F., Barbacioru C., Nordman E., Bao S., Lee C., Wang X., Tuch B.B., Heard E., Lao K., Surani M.A. (2011). Deterministic and stochastic allele specific gene expression in single mouse blastomeres. PLoS One.

[143] Ohnishi Y., Huber W., Tsumura A., Kang M., Xenopoulos P., Kurimoto K., Oles A.K., Arauzo-Bravo M.J., Saitou M., Hadjantonakis A.K. (2014). Cell-to-cell expression variability followed by signal reinforcement progressively segregates early mouse lineages. Nat. Cell Biol..

[144] Sulston J.E., Horvitz H.R. (1977). Post-embryonic cell lineages of the nematode, Caenorhabditis elegans. Dev. Biol..

[145] Bidard F.C., Weigelt B., Reis-Filho J.S. (2013). Going with the flow: from circulating tumor cells to DNA. Sci. Transl. Med..

[146] Klein C.A., Seidl S., Petat-Dutter K., Offner S., Geigl J.B., Schmidt-Kittler O., Wendler N., Passlick B., Huber R.M., Schlimok G. (2002). Combined transcriptome and genome analysis of single micrometastatic cells. Nat. Biotechnol..

[147] Dai L., Guinea M.C., Slomiany M.G., Bratoeva M., Grass G.D., Tolliver L.B., Maria B.L., Toole B.P. (2013). CD147-dependent heterogeneity in malignant and chemoresistant properties of cancer cells. Am. J. Pathol..

[148] Schwartz S., Agarwala S.D., Mumbach M.R., Jovanovic M., Mertins P., Shishkin A., Tabach Y., Mikkelsen T.S., Satija R., Ruvkun G. (2013). High-resolution mapping reveals a conserved, widespread, dynamic mRNA methylation program in yeast meiosis. Cell.

[149] Fustin J.M., Doi M., Yamaguchi Y., Hida H., Nishimura S., Yoshida M., Isagawa T., Morioka M.S., Kakeya H., Manabe I. (2013). RNA-methylation-dependent RNA processing controls the speed of the circadian clock. Cell.

[150] Nishikura K. (2010). Functions and regulation of RNA editing by ADAR deaminases. Annu. Rev. Biochem..

[151] Tilgner H., Raha D., Habegger L., Mohiuddin M., Gerstein M., Snyder M. (2013). Accurate identification and analysis of human mRNA isoforms using deep long read sequencing. G3 (Bethesda).

[152] Armour C.D., Castle J.C., Chen R., Babak T., Loerch P., Jackson S., Shah J.K., Dey J., Rohl C.A., Johnson J.M. (2009). Digital transcriptome profiling using selective hexamer priming for cDNA synthesis. Nat. Methods.

[153] Derrien T., Johnson R., Bussotti G., Tanzer A., Djebali S., Tilgner H., Guernec G., Martin D., Merkel A., Knowles D.G. (2012). The GENCODE v7 catalog of human long noncoding RNAs: analysis of their gene structure, evolution, and expression. Genome Res..

[154] Tang F., Hajkova P., Barton S.C., O'Carroll D., Lee C., Lao K., Surani M.A. (2006). 220-plex microRNA expression profile of a single cell. Nat. Protoc..

[155] Tang F., Hajkova P., Barton S.C., Lao K., Surani M.A. (2006). MicroRNA expression profiling of single whole embryonic stem cells. Nucleic Acids Res..

[156] Kozomara A., Griffiths-Jones S. (2014). miRBase: annotating high confidence microRNAs using deep sequencing data. Nucleic Acids Res..

[157] Gupta V., Poss K.D. (2012). Clonally dominant cardiomyocytes direct heart morphogenesis. Nature.

[158] Blainey P.C. (2013). The future is now: single-cell genomics of bacteria and archaea. FEMS Microbiol. Rev..

[159] Rinke C., Schwientek P., Sczyrba A., Ivanova N.N., Anderson I.J., Cheng J.F., Darling A., Malfatti S., Swan B.K., Gies E.A. (2013). Insights into the phylogeny and coding potential of microbial dark matter. Nature.

[160] Shi Y., Tyson G.W., DeLong E.F. (2009). Metatranscriptomics reveals unique microbial small RNAs in the ocean's water column. Nature.

[161] Branton D., Deamer D.W., Marziali A., Bayley H., Benner S.A., Butler T., Di Ventra M., Garaj S., Hibbs A., Huang X. (2008). The potential and challenges of nanopore sequencing. Nat. Biotechnol..

[162] Schneider G.F., Dekker C. (2012). DNA sequencing with nanopores. Nat. Biotechnol..

[163] Nivala J., Marks D.B., Akeson M. (2013). Unfoldase-mediated protein translocation through an alpha-hemolysin nanopore. Nat. Biotechnol..

[164] Eisenstein M. (2012). Oxford Nanopore announcement sets sequencing sector abuzz. Nat. Biotechnol..

[165] Ayub M., Bayley H. (2012). Individual RNA base recognition in immobilized oligonucleotides using a protein nanopore. Nano Lett..

[166] Cracknell J.A., Japrung D., Bayley H. (2013). Translocating kilobase RNA through the Staphylococcal alpha-hemolysin nanopore. Nano Lett..

[167] Ayub M., Hardwick S.W., Luisi B.F., Bayley H. (2013). Nanopore-based identification of individual nucleotides for direct RNA sequencing. Nano Lett..

[168] Hardwick S.W., Chan V.S., Broadhurst R.W., Luisi B.F. (2011). An RNA degradosome assembly in Caulobacter crescentus. Nucleic Acids Res..

[169] Ying Y.-L., Zhang J., Meng F.-N., Cao C., Yao X., Willner I., Tian H., Long Y.-T. (2013). A Stimuli-Responsive Nanopore Based on a Photoresponsive Host-Guest System. Scientific Reports 3.

[170] Westermann A.J.1, Gorski S.A., Vogel J. (2012). Dual RNA-seq of pathogen and host. Nat. Rev. Microbiol..

